# A pilot randomized controlled trial of neuromodulation-augmented balance training in people with multiple sclerosis: STIM-Balance Protocol

**DOI:** 10.1371/journal.pone.0346491

**Published:** 2026-04-16

**Authors:** Shirin Tajali, Jillian Scandiffio, Yasma Ali-Hassan, Derrick Lim, Rayna Ghosh, Sharmini Atputharaj, Kristin Musselman, Robert Simpson, Kei Masani

**Affiliations:** 1 The KITE Research Institute, Toronto Rehabilitation Institute – University Health Network, Toronto, Ontario, Canada; 2 Rehabilitation Sciences Institute, University of Toronto, Toronto, Ontario, Canada; 3 Institute of Biomedical Engineering, University of Toronto, Toronto, Ontario, Canada; 4 Department of Physical Therapy and Rehabilitation Sciences Institute, University of Toronto, Toronto, Ontario, Canada; PLOS: Public Library of Science, UNITED KINGDOM OF GREAT BRITAIN AND NORTHERN IRELAND

## Abstract

**Background:**

Impairments in balance control and falls are common problems for people with multiple sclerosis (PwMS), resulting in mobility limitations and reduced participation. Non-invasive neuromodulation techniques such as functional electrical stimulation (FES) and transcutaneous spinal stimulation (TSS) have revealed promising results in improving motor functions in other neurological populations; however, their effects during task-specific balance training have not been investigated in PwMS.

**Objective:**

To evaluate the feasibility, acceptability, safety, and preliminary clinical efficacy of neuromodulation-augmented balance training programs on balance, mobility, and neuroplasticity in PwMS (ClinicalTrials.gov Identifier: NCT07174973).

**Methods:**

Twenty-four ambulatory PwMS will be randomly assigned into three groups: (1) visual feedback balance training (VFBT) with sham stimulation, (2) VFBT with active (closed-loop) FES for the ankle muscles and sham TSS, and (3) VFBT with active FES and active (open-loop sub-motor-threshold) TSS at the lumbosacral enlargement. Participants in each group will complete 12 training sessions over six weeks. Feasibility, safety, and acceptability will be assessed through recruitment and adherence metrics, adverse-event monitoring, and semi-structured interviews guided by the Technology Acceptance Model questionnaire-2. Performance-based measures of balance, mobility, and walking speed, as well as patient-reported outcomes of balance confidence, walking ability, and fear of falling will be recorded to assess the preliminary efficacy. Modulation in neural pathways excitability will be quantified by recording motor evoked potentials and spinal motor evoked potentials.

**Conclusion:**

Findings will help to determine whether neuromodulation-augmented balance training is feasible, safe, and acceptable for PwMS and will guide the design of a future fully powered RCT.

## 1. Introduction

More than 75% of people with multiple sclerosis (PwMS) exhibit some degree of postural imbalance, even in the early stages of the disease [1–6]. Deficits in balance control are considered significant risk factors for falls, resulting in diminished physical activity and participation [1–6]. Although evidence supports the effectiveness of exercise-based training programs in PwMS to improve balance [2,3,7,8], these improvements are not sufficient to meaningfully mitigate fall risk [7]. Furthermore, the evidence is more limited in PwMS in comparison to other neurological populations, such as those with stroke and Parkinson’s disease, where more systematic reviews and clinical guidelines have been developed to improve mobility and prevent falls [9–15]. This highlights the need to develop innovative, task-specific, challenging rehabilitation programs to improve mobility and reduce falls in PwMS

The advent of rehabilitation technologies has facilitated the incorporation of interactive, game-oriented components into virtual and augmented training settings [16–22]. These methods have provided motivating and task-specific environments for people with neurological conditions, including spinal cord injury (SCI), stroke, and MS to practice motor skills [16–22]. The growing body of evidence also indicates that integrating non-invasive neuromodulation techniques such as functional electrical stimulation (FES) and transcutaneous spinal stimulation (TSS) into neurorehabilitation protocols can further improve motor function and promote neuroplasticity in people with neurological conditions [23–30]. FES can activate paralyzed or weak muscles and facilitate movements by selectively stimulating superficial motor nerves and/or muscles, however, it recruits motor units in a non-physiological pattern, which can result in rapid muscle fatigue [31–33]. TSS can activate multiple muscles by stimulating dorsal roots and modulating excitability across multiple spinal segments, potentially resulting in less muscle fatigue [34,35]. However, its ability to produce direction-specific movements required for balance corrections is limited compared to FES [28–30,36].

Our team has developed a balance training platform that integrates visual feedback balance training (VFBT) with closed-loop FES for ankle muscles [24,37,38]. We have tested the feasibility and preliminary efficacy of this protocol in individuals with incomplete SCI who had difficulty in balance but were still able to stand without support [24,39]. However, for individuals with lower levels of function, particularly those with knee and hip impairments, FES for the ankle muscles may be insufficient to provide the postural support necessary for effective balance training [27,40,41]. Although proximal muscles could be stimulated by additional FES channels, this would significantly increase the system’s complexity. Rather, TSS offers a more effective way to concurrently modulate excitability across several spinal segments [28–30,36], and to augment multi-muscle activation without adding technical burden. This synergistic interaction is supported by preliminary results from our research group in people with incomplete SCI, showing greater functional benefits when TSS is administered concurrently with closed-loop FES (TSS + FES) during balance tasks [42,43].

Given the novelty of this combined neuromodulation approach and the lack of feasibility data in PwMS, a pilot randomized controlled trial (RCT) is warranted before designing a fully powered RCT. Therefore, the main purpose of this study is to assess the feasibility, acceptability, safety, and adherence of neuromodulation-augmented balance training interventions in PwMS. The second purpose is to investigate the preliminary clinical efficacy on balance performance, mobility, balance confidence, fear of falling and neural excitability. We hypothesize that the intervention will be feasible and acceptable with minimal adverse events, and that the combined neuromodulation group (TSS + FES) will show greater functional and neurophysiological improvements than FES-only or sham stimulation groups.

## 2. Methods & materials

### 2.1. Participants & eligibility criteria

This multi-method study includes a pilot RCT to evaluate feasibility, acceptability, safety and preliminary efficacy of this novel neuromodulation-augmented balance training program and a qualitative descriptive study to capture patients’ experiences and perspectives [44]. This study will take place at the Lyndhurst Centre, a tertiary rehabilitation hospital, and has been approved by the University Health Network Research Ethics Board (Approval #25–5549, Version 5, December 29, 2025) and is registered at ClinicalTrials.gov (Identifier: NCT07174973). A formal sample size calculation is not required, as this is a pilot RCT [45]. We plan to recruit 24 participants (eight in each group), based on our previous studies in people with SCI using non-invasive neuromodulation.

For recruitment, we will use multiple strategies, including clinicians’ referrals, posting flyers at outpatient MS clinics, community MS programs, and existing research registries/databases (MS Canada). Participants will be allowed to continue their usual medical care throughout the study period. Written informed consent will be obtained from all eligible participants prior to baseline assessment. Given the small sample size, single-site design, and low-risk nature of the interventions, a formal independent data monitoring committee will not be required for this pilot RCT. Participant safety and study conduct will be monitored by the principal investigator, and any adverse events will be reported to the University Health Network Research Ethics Board. In addition, ongoing monitoring will occur through regular research team meetings to review recruitment progress, protocol adherence, intervention fidelity, data completeness, and adverse events.

**Fig 1:**
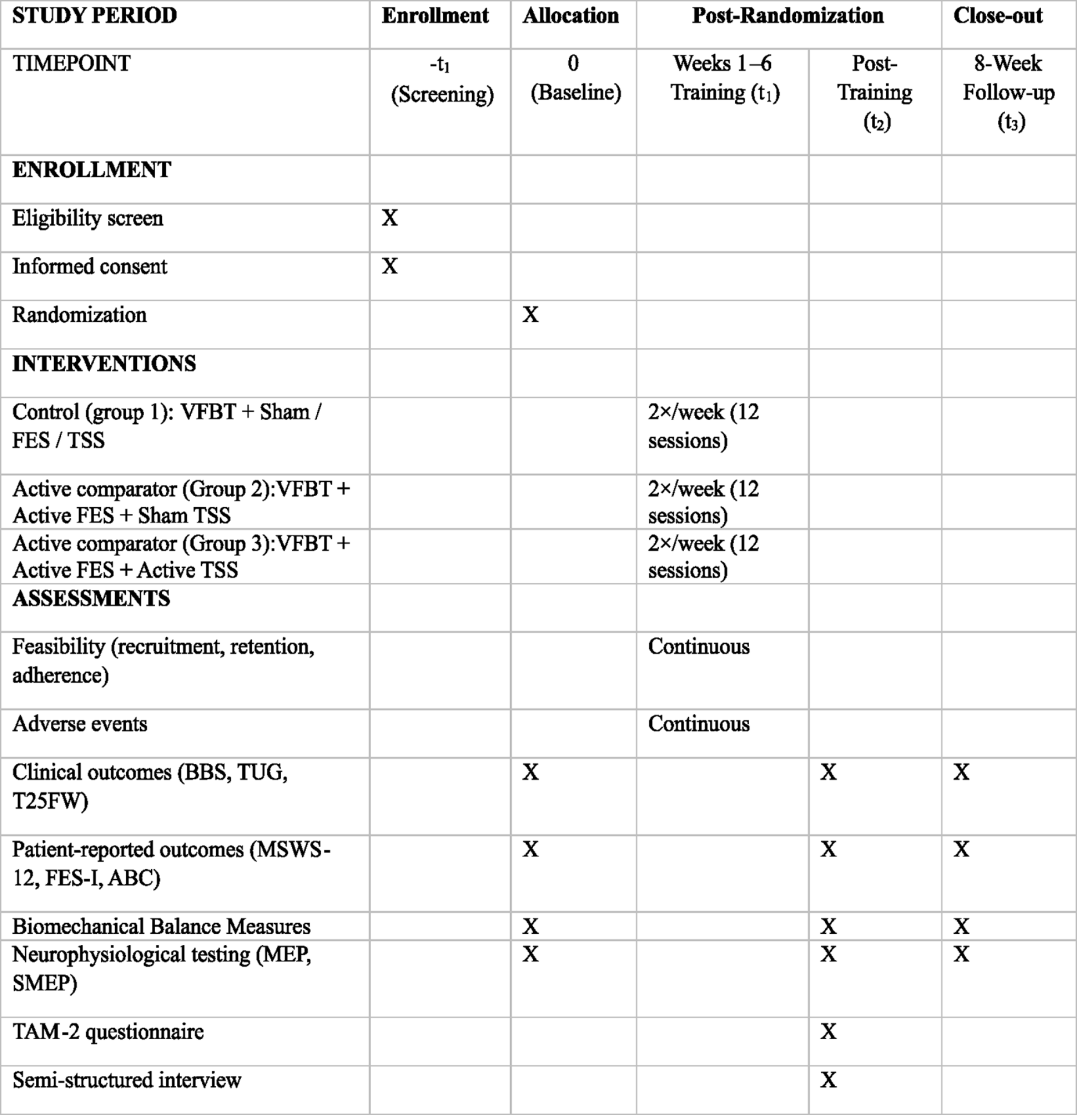
SPIRIT Timeline: Schedule of enrollment, interventions, and assessments. Legend: VFBT: Visual Feedback Balance Training, FES: Functional Electrical Stimulation, TSS: Transcutaneous Spinal Stimulation, TAM-2: Technology Acceptance Model-2, BBS: Berg Balance Scale; T25FW: Timed 25-Foot Walk, TUG: Timed Up and Go, ABC: Activities-specific Balance Confidence Scale, MSWS-12: 12-item Multiple Sclerosis Walking Scale, FES-I = Falls Efficacy Scale-International; GRC: Global Rating of Change, MEPs: Motor Evoked Potentials, SMEPs: Spinal Motor Evoked Potentials.

Fig 1 shows the SPIRIT Timeline for the study, Table 1 summarizes the inclusion and exclusion criteria, and Table 2 shows the template for Intervention Description and Replication (TIDieR) checklist.

**Table 1:** Inclusion and exclusion criteria.

Criteria	
Inclusion	Confirmed diagnosis of MS based on the revised McDonald criteria Age 18–65 years Ability to walk at least 100 meters with or without an assistive device Self-reported balance difficulties during daily activities
Exclusion	Uncorrected visual impairments that interfere with balance testing Cognitive deficits limiting comprehension of study procedures MS relapse within the past 30 days Presence of other neurological or orthopedic conditions affecting balance Recent participation in structured rehabilitation within the past month Lower-limb botulinum toxin injections within the past three months Known peripheral nerve injuries Contraindications to electrical stimulation (e.g., implanted metal/electronic devices, pacemakers) Contraindications to TMS (e.g., seizure history, metallic implants in the head). Pregnancy

**Table 2:** The TIDieR checklist.

1. Brief Name	Visual Feedback Balance Training (VFBT) with (1) sham neuromodulation, (2) closed-loop functional electrical stimulation (FES), and (3) combined closed-loop FES and transcutaneous spinal stimulation (TSS + FES).
2. Why	To improve balance, mobility, and neuroplasticity in PwMS through task-specific balance training combined by neuromodulation. VFBT facilitates patient’s engagement and motor learning through real-time feedback; FES helps to activate ankle muscles and produces direction-specific ankle torques during balance training; TSS modulates spinal excitability to augment the effects of FES and supra-spinal control by voluntary contribution.
3. Who	Interventions will be delivered by trained clinical researchers with experience in balance rehabilitation and non-invasive neuromodulation using standardized procedures.
4. When	The intervention will be delivered to ambulatory PwMS who are clinically stable (absence of an MS relapse within the last 30 days)
5. What	**Materials** **VFBT:** Wii Balance Board (Nintendo, Japan), monitor display, ceiling-mounted safety harness. **FES:** stimulator: Myndsearch, MyndTec Inc., Mississauga, ON, Canada, surface electrodes (5 × 5 cm for dorsiflexors; 5 × 10 cm for plantarflexors). **TSS:** stimulator: Pajunk® GmbH Medizinprodukte, Geisingen, Germany, surface electrodes (5 × 10 cm) positioned over T10–L2 with anterior reference electrodes (5 × 5 cm) placed over the abdomen medial to the anterior superior iliac spine. **Procedures** Participants will perform static and dynamic standing balance tasks with real-time visual feedback regarding their postural sway. Tasks will include target tracking, ellipse tracing, color-matching, and hunting games. Neuromodulation (active or sham) is delivered concurrently according to group allocation.
6. Who	Interventions will be delivered by experienced clinical researchers who are trained in balance rehabilitation and using non-invasive neuromodulation.
7. How	In-person, one-on-one supervised sessions.
8. Where	University Health Network, Lyndhurst Rehabilitation Hospital.
9. How Much	12 sessions over 6 weeks (2 sessions/week). Each session will last approximately 90 minutes, consisting of setup, warm-up, 60 minutes of active training, and a cool-down.
10. How challenging	The balance training is designed to be progressively challenging and tailored to each participant’s abilities. VFBT difficulty will be increased by changing center-of-pressure excursion range, movement speed, and duration of the game. Warm-up balance exercises will be progressed from standing on a rigid, stable surface to standing on unstable surfaces, and eventually walking-based balance tasks.
11. Progression/Regression	Balance task difficulty will be progressed by increasing center of pressure excursion range, task speed, and the duration of each game.
12. Personalization	FES stimulation intensities will be individualized based on motor threshold and maximal tolerable intensity; balance task difficulty will be adjusted based on the participant’s baseline balance calibration by testing limits of stability; TSS will be delivered at a sub-motor threshold level
13. Protocol Deviation	No planned modifications. Any protocol deviations, such as changes in the stimulation intensity due to discomfort or fatigue will be documented.
14. How Well	Session attendance will be tracked; stimulation parameters will be recorded at each session. Adherence rates, and any protocol deviations will be reported
15. Harms	Adverse events will be monitored at each session (e.g., skin irritation, fatigue, dizziness, discomfort).

PwMS: people with multiple sclerosis, VFBT: visual feedback balance training, FES: functional electrical stimulation, TSS: transcutaneous spinal cord stimulation, TSS + FES: combined transcutaneous spinal cord stimulation and functional electrical stimulation, MEPs: motor evoked potentials, SMEPs: spinal motor evoked potentials, TAM: Technology Acceptance Model, COP: center of pressure, FU: follow-up.

### 2.2. Randomization, allocation, and blinding

Participants will be randomly assigned into three groups following baseline assessment using a computer-generated covariate-adaptive algorithm, including two variables: (1) gender, and (2) baseline postural control using the forward reach test of the Berg Balance Scale (BBS). To ensure balanced allocation of people with different levels of function, participants with scores of 0–2 in the forward reach test (i.e., reaching forward ≤2 inches) will be categorized as having low postural control, while those with scores of 3–4 (i.e., reaching forward ≥5 inches) will be categorized as having high postural control. Outcome assessors conducting clinical assessment will be blinded to group assignment. The neurophysiological assessments will be conducted by one of the therapists delivering the intervention and will therefore not be blinded. Since the neurophysiological assessment involves standardized instrumentation, they are less susceptible to assessor bias [46]. Participants will remain blinded through the use of sham stimulation.

### 2.3. Treatment groups

**Fig 2:**
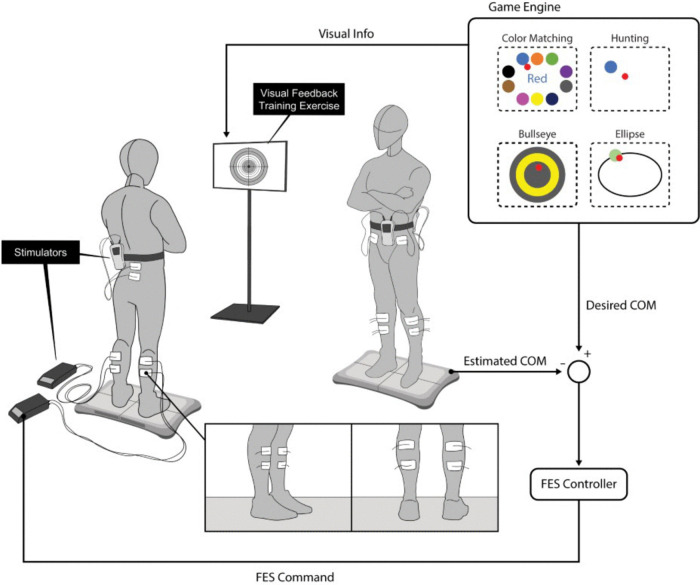
Visual feedback balance training set up with functional electrical stimulation (FES) and transcutaneous spinal stimulation (TSS).

A standardized program of balance exercises and VFBT will be provided to participants across groups twice a week for six weeks, totaling twelve sessions. VFBT is a task-specific training approach in which participants receive real-time visual feedback on their balance performance during static and dynamic standing tasks [37,38,47]. Each training session will last approximately 90 minutes, including a warm-up period with light aerobic activity (e.g., marching in place), lower-limb stretching, and low-intensity controlled weight shifting exercises customized to each participant’s functional level, followed by 60 minutes of active training. The conceptual framework of our setup, which includes VFBT with FES & TSS, is depicted in Fig 2. For VFBT, participants will stand in front of a monitor while a Wii-Balance Board (Nintendo, Japan) records center of pressure (COP) displacement, which is displayed on the screen as a cursor to guide postural adjustments. Training includes interactive games such as target tracking, ellipse tracing, color-matching, and hunting [37,38,47]. Participants will be encouraged not to use assistive devices except when necessary for safety in the event of difficulty with independent standing. A ceiling-mounted harness will be provided to each participant to prevent falls while allowing movements during the training.

#### 2.3.1. Group 1: VFBT with Sham FES + Sham TSS.

Participants in this group will receive the sham simulation during VFBT. One electrode (5 × 10 cm) will be positioned over the thoracolumbar spine between T10-L2, and two inter-connected reference electrodes (5 × 5 cm) will be placed over the abdomen, medial to the anterior superior iliac spine. For FES, surface electrodes will be placed over the dorsi-flexors (5 × 5 cm) and the plantar-flexors (5 × 10 cm) on both sides. During the first 30 seconds of each session, there will be brief sensory-level stimulation, which will be dropped to 0 mA.

#### 2.3.2. Group 2: VFBT with Active FES + Sham TSS.

Participants in this group will receive active FES for ankle muscles and sham TSS during VFBT. At the start of each session, motor threshold and maximum tolerable stimulation intensities will be determined for each muscle. FES will be delivered at a frequency of 30 Hz with a pulse width of 0.3 ms (MyndSearch, MyndTec Inc., Mississauga, ON, Canada). The closed-loop FES activates ankle muscles in response to real-time feedback from the Wii-Balance Board, which monitors the user’s COP. The filtered COP represents the body’s center of mass (COM). The body’s COM is continually monitored and the level of stimulation adjusted accordingly on a moment-to-moment basis, mimicking intact postural control. Stimulation intensity will vary between the motor threshold and 90% of the maximal tolerable intensity to facilitate the intended movements during balance tasks [37,38,47]. Sham TSS will be administered via a brief sensory-level ramp, then decreased to 0 mA, similar to group 1.

#### 2.3.3. Group 3: VFBT with Active FES +Active TSS.

Participants in this group will receive the same active FES protocol described above, plus active TSS which will be applied using surface electrodes positioned over the T10–L2, with anterior electrodes placed over the abdomen medial to the anterior superior iliac spine (Fig 2). Stimulation will be delivered continuously at a sub-motor threshold at 30 Hz using biphasic, 1 ms pulse width (Stim2Go, Pajunk® GmbH, Germany) during VFBT.

### 2.4. Treatment outcomes

The study outcomes have been categorized into feasibility, acceptability, safety, efficacy, and neurophysiological outcomes quantifying neuroplasticity. Below is a full explanation of each outcome category.

#### 2.4.1. Feasibility outcomes.

Feasibility outcomes will include recruitment rate, retention rate, and adherence [48]. Recruitment rate refers to the percentage of eligible participants who enrolled relative to the number screened. Retention is defined as the proportion of participants who completed all three assessment time points, and adherence is measured by how many of the 12 training sessions each participant completed.

#### 2.4.2. Acceptability outcomes.

The Technology Acceptance Model 2 (TAM-2) questionnaire will be used immediately after training to capture patients’ perspectives of perceived usefulness of the technology for training [49,50]. TAM-2 includes questions reflecting different domains of technology acceptance, including behavioral intention (e.g., intention to use again, social influence), perceived ease of use (e.g., enjoyment, comfort/safety), perceived usefulness (e.g., goal attainment, impact on daily life), and usage behavior (e.g., real-world integration, user preferences, individualization) [49]. Participants will also take part in semi-structured qualitative interviews guided by a previously developed framework based on TAM-2 [49]. The guide consists of open-ended questions and encourages participants to share their thoughts and experience regarding perceived benefits of the training program across different domains aligned with the TAM-2 framework (behavioral intention, perceived ease of use, usefulness, and usage behavior) [49].

#### 2.4.3. Safety outcomes.

All adverse events will be recorded and reported throughout the study period. Adverse events may include skin irritation, muscle soreness, discomfort, fatigue, dizziness, risk of loss of balance, or any unexpected pain. If participants request to withdraw from the trial, the intervention will be discontinued, and they will be withdrawn from the trial without penalty or impact on their usual medical care. All adverse events and the actions taken to address them will be reported to the Review Ethics Board. Participants will receive medical treatment at no cost to them in accordance with institutional policies in the event of an adverse event.

#### 2.4.4. Efficacy outcomes (Exploratory).

The clinical performance-based tests, biomechanical balance tests, and patient-reported outcomes will be examined at baseline, immediately post-training, and at 8 weeks post-training. Performance-based tests will include the BBS, Timed Up and Go (single- and dual-task), and the Timed 25-Foot Walk (T25FW). The BBS is a 14-item performance-based measure of functional balance that has demonstrated excellent reliability and validity in PwMS [51,52]. The TUG assesses functional mobility and dynamic balance and has demonstrated good reliability and sensitivity to change in MS, including under dual-task conditions [51,52]. The T25FW is a commonly used measure of walking speed that has shown to be reliable and valid in PwMS [51,52]. We will additionally test static and dynamic balance performance using a force plate to measure sway characteristics and limits of stability.

Patient-reported outcomes will include MS Walking Scale-12 (self-reported measure of walking abilities and limitations), Falls Efficacy Scale–International (concern about falling during daily activities), and the Activities-specific Balance Confidence Scale (balance confidence during daily activities). All of these patient-reported outcomes have demonstrated good reliability and validity in PwMS [53–56]. A global rating of change scale will be used to measure participants’ overall improvement immediately and at 8 weeks post-training compared to the baseline. The scale consists of a 7-point Likert scale ranging from very much worse to very much improved [57,58].

#### 2.4.5. Biomechanical balance measures.

Participants will be asked to stand quietly on a force plate for 100 seconds under two conditions: eyes open, and COP displacements will be recorded. For the dynamic balance test, they have to lean as far as possible in eight different directions without losing balance and maximal COP excursion will be quantified.

#### 2.4.6. Neurophysiological outcomes.

**Fig 3:**
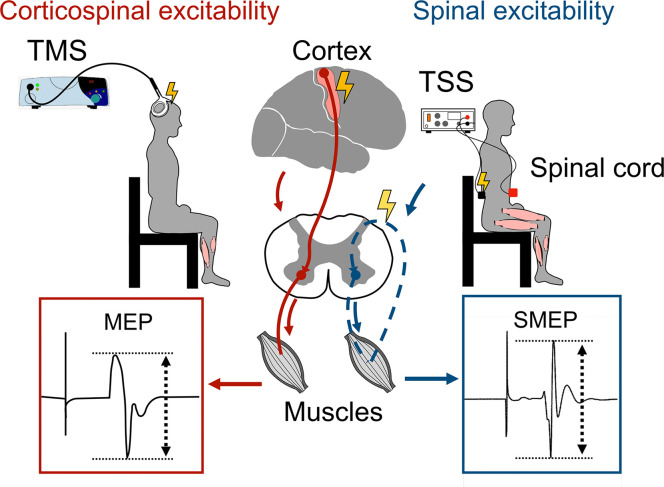
Measurement of corticospinal and spinal excitability. Motor evoked potentials (MEPs) are evoked by transcranial magnetic stimulation (TMS), and spinal motor evoked potentials (SMEPs) are evoked by transcutaneous spinal stimulation (TSS).

To measure corticospinal excitability of the leg muscles, motor evoked potentials (MEPs) will be measured using TMS with a double-cone coil (MEGA TMS system, Soterix Medical Inc., USA). Fig 3 shows the testing position for measuring MEPs. Electromyography (EMG) electrodes (Kendall TM Medi-Trace ® Mini 130 Foam Electrodes, Neurotronics, NSW, Australia) will be placed over the soleus and tibialis muscles on the more affected side. In case of symmetrical lower-limb involvement, defined as no difference in single-leg stance time between the two sides on the BBS, we will record from the dominant side. EMG signals will be amplified (×500) and band-pass filtered from 10 Hz to 1 kHz (Bortec Biomedical, AB, Canada).

The coil will be placed over the primary motor cortex, and the hotspots will be searched manually by finding a spot that can elicit the largest response in the tibialis anterior with the lowest intensity. The location of the hotspot will be stored in the brain navigation system (Neuronavigation, Soterix Medical Inc.) to ensure a consistent stimulation location above the motor cortex at each time point [59]. Then, the resting motor threshold will be determined as the intensity of magnetic stimulation required to evoke MEPs with a peak-to-peak amplitude of 50 micro-V in at least 5 out of 10 consecutive trials. MEPs will subsequently be recorded at five stimulation intensities corresponding to 100%, 110%, 120%, 130%, and 140% of the session-specific RMT, with three trials collected at each intensity to characterize the stimulus–response relationship. To obtain a stable estimate of corticospinal excitability, an additional ten MEPs will be recorded at 120% RMT. To normalize MEP amplitudes, we will measure the maximal motor wave (M-max) in soleus and tibialis anterior after TMS testing. Soleus and tibialis anterior M-max will be measured by applying maximal single square pulses to the tibial nerve in the popliteal fossa and the common peroneal nerve around the head of the fibula, respectively.

Spinal motor evoked potentials (SMEPs) will be assessed with double-pulse stimulation (Digitimer, Model DS7A, Welwyn Garden City, UK) delivered over L1-L2 (two monophasic pulses with 1ms pulse width and a 50 ms inter-stimulus interval). Surface EMG will be recorded from the more affected side or dominant side in case of symmetrical involvement from four lower-limb muscles: soleus, tibialis anterior, rectus femoris, and biceps femoris. The same amplifier will be used to both amplify and band-pass-filter the data, as described above. Recruitment curves will be generated by increasing stimulation intensity from 5 mA to the participant’s maximal tolerable level in 5-mA increments. At each intensity, five paired-pulse stimulations were delivered with 5-second inter-trial intervals.

### 2.5. Assessment timeline

As outlined in Table 1, feasibility and safety outcomes will be measured throughout the study. Acceptability outcomes will be measured post-training immediately. Clinical outcomes will be measured at three time points: (1) baseline, (2) immediately post-training (two or three days after completing the 12 sessions), and (3) eight-week post-training. There will be two visits at each time point: one for clinical and mobility assessments, and the other for neurophysiological testing and an interview. Safety outcomes will be measured throughout the study.

### 2.6. Data management & analysis

Study data will be entered into a secure, password-protected electronic database by the University Health Network. Participants will be assigned a unique study identification number, and identifying information will be stored separately from research data. Electronic data will be stored on the secure institutional server, and paper records will be stored in locked cabinets in the secured research office. Feasibility outcomes (recruitment, retention, adherence, completion rates) will be summarized descriptively using means, standard deviations, frequencies, and percentages. Regarding TAM-2, items will be grouped into predetermined domains, and scores within each domain will be averaged to generate domain-specific scores. Conventional content analysis will be used to analyze the interview transcripts, given the exploratory nature of the study and limited research in this area. Interviews will be recorded, transcribed, and analyzed inductively by two members of the research team. The researchers will independently review the same transcripts, then meet to identify codes and create a preliminary codebook. This process will be repeated until a final codebook is created, and then the final codebook will be applied to the transcripts. Next, the research team will meet to review the codebook and group the codes, which will then be organized into categories and themes [24,39,61]. Adverse events will be reported descriptively by frequency and type.

All statistical analyses will be performed using SPSS (IBM SPSS, Chicago, USA) for the available data. A two-way repeated-measures ANOVA (group × time) will be conducted to analyze changes in clinical and neurophysiological outcomes across three study groups and time. The effect sizes with 95% confidence intervals will be reported, and the proportion of missing data (if applicable) will be described to inform the design and sample size calculation of a future fully powered trial.

### 2.7. Knowledge translation plan

Findings from this study will be widely disseminated to relevant knowledge users, including PwMS and their caregivers, rehabilitation clinicians, rehabilitation researchers, and organizations engaged in MS care, through peer-reviewed publications, relevant conferences, and workshops. We anticipate that the findings will be disseminated across two to three publications: one publication will focus on feasibility outcomes, the second publication will report preliminary clinical and neurophysiological outcomes, and the third publication will focus on the qualitative findings to provide more details regarding the participant experiences, perceived barriers, and facilitators of the intervention, and suggestions for intervention refinement.

## 3. Discussion

Non-invasive neuromodulation techniques, including FES and TSS, have shown efficacy in improving walking and upper limb motor functions when coupled with active task-specific training in people with neurological conditions [23,29,30,33,62–65]. However, their potential effects as complementary interventions to improve balance in PwMS have yet to be investigated. This pilot RCT aims to fill this gap by assessing the feasibility of integrating technology specifically non-invasive neuromodulation and task-specific balance training via VFBT and evaluating its effects on balance, mobility, and neuroplasticity in PwMS. Running this pilot study will help to identify methodological, logistical, and implementation-related challenges prior to conducting larger RCTs [66–69].

In addition to feasibility and acceptability outcomes, the study includes a battery of clinical and neurophysiological assessments. These exploratory measures will help to determine whether neuromodulation-augmented VFBT has the potential to improve balance, mobility, balance confidence, and fear of falling. Neurophysiological assessment will help to provide preliminary insight into whether changes in corticospinal and spinal excitability are associated with functional improvements. These mechanistic measures are recommended in neuromodulation trials to identify the underlying neural mechanisms associated with recovery and guide the design of future studies [28,60].

The methodological strengths of this study are consistent with high-quality trials. The randomized, sham-controlled, and assessor-blinded design reduces bias, and covariate-adaptive randomization helps to normalize baseline differences and reduce heterogeneity across groups [70–72]. Furthermore, incorporation of semi-structured interviews offers a more in-depth understanding of participants’ experiences and perspectives, which is of great importance when considering to integrate neuromodulation techniques into rehabilitation settings. Preliminary clinical outcomes will help to calculate the effect size and variance necessary to plan a definitive RCT.

## References

[1] Cameron MH, Nilsagard Y. Balance, gait, and falls in multiple sclerosis. Handbook of clinical neurology. 2018. p. 237–50. 10.1016/B978-0-444-63916-5.00015-X 30482317

[2] Monjezi S, Negahban H, Tajali S, Yadollahpour N, Majdinasab N. Effects of dual-task balance training on postural performance in patients with Multiple Sclerosis: a double-blind, randomized controlled pilot trial. Clin Rehabil. 2017;31(2):234–41. doi: 10.1177/0269215516639735 27006419

[3] Corrini C, Gervasoni E, Perini G, Cosentino C, Putzolu M, Montesano A, et al. Mobility and balance rehabilitation in multiple sclerosis: A systematic review and dose-response meta-analysis. Mult Scler Relat Disord. 2023;69:104424. doi: 10.1016/j.msard.2022.104424 36473240

[4] Sosnoff JJ, Socie MJ, Boes MK, Sandroff BM, Pula JH, Suh Y, et al. Mobility, balance and falls in persons with multiple sclerosis. PLoS One. 2011;6(11):e28021. doi: 10.1371/journal.pone.0028021 22132196 PMC3222674

[5] Hoang PD, et al. Neuropsychological, balance, and mobility risk factors for falls in people with multiple sclerosis: a prospective cohort study. Archives of Physical Medicine and Rehabilitation. 2014;95(3):480–6. 24096187 10.1016/j.apmr.2013.09.017

[6] Tajali S, Shaterzadeh-Yazdi M-J, Negahban H, van Dieën JH, Mehravar M, Majdinasab N, et al. Predicting falls among patients with multiple sclerosis: Comparison of patient-reported outcomes and performance-based measures of lower extremity functions. Mult Scler Relat Disord. 2017;17:69–74. doi: 10.1016/j.msard.2017.06.014 29055478

[7] Gunn H, Markevics S, Haas B, Marsden J, Freeman J. Systematic Review: The Effectiveness of Interventions to Reduce Falls and Improve Balance in Adults With Multiple Sclerosis. Arch Phys Med Rehabil. 2015;96(10):1898–912. doi: 10.1016/j.apmr.2015.05.018 26070975

[8] Martino Cinnera A, Bisirri A, Leone E, Morone G, Gaeta A. Effect of dual-task training on balance in patients with multiple sclerosis: A systematic review and meta-analysis. Clin Rehabil. 2021;35(10):1399–412. doi: 10.1177/02692155211010372 33874763

[9] Hornby TG, Reisman DS, Ward IG, Scheets PL, Miller A, Haddad D, et al. Clinical Practice Guideline to Improve Locomotor Function Following Chronic Stroke, Incomplete Spinal Cord Injury, and Brain Injury. J Neurol Phys Ther. 2020;44(1):49–100. doi: 10.1097/NPT.0000000000000303 31834165

[10] Coelho DB, de Oliveira CEN, Guimarães MVC, Ribeiro de Souza C, Dos Santos ML, de Lima-Pardini AC. A systematic review on the effectiveness of perturbation-based balance training in postural control and gait in Parkinson’s disease. Physiotherapy. 2022;116:58–71. doi: 10.1016/j.physio.2022.02.005 35550488

[11] Shen X, Wong-Yu ISK, Mak MKY. Effects of Exercise on Falls, Balance, and Gait Ability in Parkinson’s Disease: A Meta-analysis. Neurorehabil Neural Repair. 2016;30(6):512–27. doi: 10.1177/1545968315613447 26493731

[12] Osborne JA, Botkin R, Colon-Semenza C, DeAngelis TR, Gallardo OG, Kosakowski H, et al. Physical Therapist Management of Parkinson Disease: A Clinical Practice Guideline From the American Physical Therapy Association. Phys Ther. 2022;102(4):pzab302. doi: 10.1093/ptj/pzab302 34963139 PMC9046970

[13] Wallin A, Johansson S, Brincks J, Dalgas U, Franzén E, Callesen J. Effects of Balance Exercise Interventions on Balance-Related Performance in People With Multiple Sclerosis: A Systematic Review and a Meta-Analysis of Randomized Controlled Trials. Neurorehabil Neural Repair. 2024;38(10):775–90. doi: 10.1177/15459683241273402 39162296 PMC11490070

[14] Biswas SS. Clinical Practice Guideline of Physiotherapy Intervention for Lower Limb on Spinal Cord Injury. 2023.

[15] Winstein CJ, Stein J, Arena R, Bates B, Cherney LR, Cramer SC, et al. Guidelines for Adult Stroke Rehabilitation and Recovery: A Guideline for Healthcare Professionals From the American Heart Association/American Stroke Association. Stroke. 2016;47(6):e98–169. doi: 10.1161/STR.0000000000000098 27145936

[16] Abou L, Malala VD, Yarnot R, Alluri A, Rice LA. Effects of Virtual Reality Therapy on Gait and Balance Among Individuals With Spinal Cord Injury: A Systematic Review and Meta-analysis. Neurorehabil Neural Repair. 2020;34(5):375–88. doi: 10.1177/1545968320913515 32270736

[17] Wu J, Zeng A, Chen Z, Wei Y, Huang K, Chen J, et al. Effects of Virtual Reality Training on Upper Limb Function and Balance in Stroke Patients: Systematic Review and Meta-Meta-Analysis. J Med Internet Res. 2021;23(10):e31051. doi: 10.2196/31051 34636735 PMC8548971

[18] Calafiore D, Invernizzi M, Ammendolia A, Marotta N, Fortunato F, Paolucci T, et al. Efficacy of Virtual Reality and Exergaming in Improving Balance in Patients With Multiple Sclerosis: A Systematic Review and Meta-Analysis. Front Neurol. 2021;12:773459. doi: 10.3389/fneur.2021.773459 34956054 PMC8702427

[19] de Araújo AVL, Neiva JF de O, Monteiro CB de M, Magalhães FH. Efficacy of Virtual Reality Rehabilitation after Spinal Cord Injury: A Systematic Review. Biomed Res Int. 2019;2019:7106951. doi: 10.1155/2019/7106951 31828120 PMC6885151

[20] Castellano-Aguilera A, Biviá-Roig G, Cuenca-Martínez F, Suso-Martí L, Calatayud J, Blanco-Díaz M, et al. Effectiveness of Virtual Reality on Balance and Risk of Falls in People with Multiple Sclerosis: A Systematic Review and Meta-Analysis. Int J Environ Res Public Health. 2022;19(21):14192. doi: 10.3390/ijerph192114192 36361069 PMC9656689

[21] Basalic EB, Roman N, Tuchel VI, Miclăuș RS. Virtual Reality Applications for Balance Rehabilitation and Efficacy in Addressing Other Symptoms in Multiple Sclerosis—A Review. Applied Sciences. 2024;14(10):4244. doi: 10.3390/app14104244

[22] Molhemi F, Mehravar M, Monjezi S, Salehi R, Negahban H, Shaterzadeh-Yazdi M-J, et al. Effects of exergaming on cognition, lower limb functional coordination, and stepping time in people with multiple sclerosis: a randomized controlled trial. Disabil Rehabil. 2023;45(8):1343–51. doi: 10.1080/09638288.2022.2060332 35443843

[23] Andreopoulou G, Busselli G, Street T, Bulley C, Safari R, van der Linden ML, et al. Is functional electrical stimulation effective in improving walking in adults with lower limb impairment due to an upper motor neuron lesion? An umbrella review. Artif Organs. 2024;48(3):210–31. doi: 10.1111/aor.14563 37259954

[24] Houston DJ, Lee JW, Unger J, Masani K, Musselman KE. Functional Electrical Stimulation Plus Visual Feedback Balance Training for Standing Balance Performance Among Individuals With Incomplete Spinal Cord Injury: A Case Series. Front Neurol. 2020;11:680. doi: 10.3389/fneur.2020.00680 32793101 PMC7390869

[25] Popovic MR, Kapadia N, Zivanovic V, Furlan JC, Craven BC, McGillivray C. Functional electrical stimulation therapy of voluntary grasping versus only conventional rehabilitation for patients with subacute incomplete tetraplegia: a randomized clinical trial. Neurorehabil Neural Repair. 2011;25(5):433–42. doi: 10.1177/1545968310392924 21304020

[26] Popovic MR, Masani K, Micera S. Functional electrical stimulation therapy: recovery of function following spinal cord injury and stroke. Neurorehabilitation Technology. Springer. 2016. p. 513–32.

[27] Tajali S, et al. The orthotic effects of different functional electrical stimulation protocols on walking performance in individuals with incomplete spinal cord injury: A case series. Topics in Spinal Cord Injury Rehabilitation. 2023;29(Supplement):142–52. 38174132 10.46292/sci23-00021S PMC10759841

[28] Tajali S, Balbinot G, Pakosh M, Sayenko DG, Zariffa J, Masani K. Modulations in neural pathways excitability post transcutaneous spinal cord stimulation among individuals with spinal cord injury: a systematic review. Front Neurosci. 2024;18:1372222. doi: 10.3389/fnins.2024.1372222 38591069 PMC11000807

[29] Megía García A, Serrano-Muñoz D, Taylor J, Avendaño-Coy J, Gómez-Soriano J. Transcutaneous Spinal Cord Stimulation and Motor Rehabilitation in Spinal Cord Injury: A Systematic Review. Neurorehabil Neural Repair. 2020;34(1):3–12. doi: 10.1177/1545968319893298 31858871

[30] Taylor C, McHugh C, Mockler D, Minogue C, Reilly RB, Fleming N. Transcutaneous spinal cord stimulation and motor responses in individuals with spinal cord injury: A methodological review. PLoS One. 2021;16(11):e0260166. doi: 10.1371/journal.pone.0260166 34793572 PMC8601579

[31] Lyu C, Panteli G, Bollheimer LC, Leonhardt S, von Platen P. Evaluation of FES-induced Muscle Fatigue and Recovery using Torque and Surface Electromyography. Annu Int Conf IEEE Eng Med Biol Soc. 2024;2024:1–4. doi: 10.1109/EMBC53108.2024.10782626 40039780

[32] Vromans M, Faghri PD. Functional electrical stimulation-induced muscular fatigue: Effect of fiber composition and stimulation frequency on rate of fatigue development. J Electromyogr Kinesiol. 2018;38:67–72. doi: 10.1016/j.jelekin.2017.11.006 29169055

[33] Masani K, Popovic MR. Functional electrical stimulation in rehabilitation and neurorehabilitation. Springer handbook of medical technology. Springer. 2011. p. 877–96.

[34] Bergquist AJ, Clair JM, Lagerquist O, Mang CS, Okuma Y, Collins DF. Neuromuscular electrical stimulation: implications of the electrically evoked sensory volley. Eur J Appl Physiol. 2011;111(10):2409–26. doi: 10.1007/s00421-011-2087-9 21805156

[35] Blazevich AJ, Collins DF, Millet GY, Vaz MA, Maffiuletti NA. Enhancing Adaptations to Neuromuscular Electrical Stimulation Training Interventions. Exerc Sport Sci Rev. 2021;49(4):244–52. doi: 10.1249/JES.0000000000000264 34107505 PMC8460078

[36] Minassian K, et al. Transcutaneous stimulation of the human lumbar spinal cord: facilitating locomotor output in spinal cord injury. Neuroscience. 2010;2010.

[37] Lee JW, Grabke E, Chow K, Musselman KE, Masani K. Development of a closed-loop controller for functional electrical stimulation therapy plus visual feedback balance training for standing balance training. Med Eng Phys. 2024;132:104238. doi: 10.1016/j.medengphy.2024.104238 39428136

[38] Houston DJ, Lee JW, Unger J, Masani K, Musselman KE. Functional Electrical Stimulation Plus Visual Feedback Balance Training for Standing Balance Performance Among Individuals With Incomplete Spinal Cord Injury: A Case Series. Front Neurol. 2020;11:680. doi: 10.3389/fneur.2020.00680 32793101 PMC7390869

[39] Houston DJ, Unger J, Lee JW, Masani K, Musselman KE. Perspectives of individuals with chronic spinal cord injury following novel balance training involving functional electrical stimulation with visual feedback: a qualitative exploratory study. J Neuroeng Rehabil. 2021;18(1):57. doi: 10.1186/s12984-021-00861-z 33794948 PMC8017659

[40] Mañago MM, et al. Contributions of ankle, knee, hip, and trunk muscle function to gait performance in people with multiple sclerosis: A cross-sectional analysis. Phys Ther. 2018;98(7):595–604. 29660080 10.1093/ptj/pzy048

[41] Citaker S, Guclu-Gunduz A, Yazici G, Bayraktar D, Nazliel B, Irkec C. Relationship between lower extremity isometric muscle strength and standing balance in patients with multiple sclerosis. NeuroRehabilitation. 2013;33(2):293–8. doi: 10.3233/NRE-130958 23949051

[42] Tajali S, K L F, Lim D, Sayenko DG, Masani K. Acute effect of a novel combined neuromodulation therapy for balance on corticospinal excitability after spinal cord injury. In IFESS 2024; 2023.

[43] Tajali SDL, Raha A, Ghosh R, Musselman KE, Sayenko DG, Masani K. Non-Invasive Neuromodulation Therapy for Enhancing Balance and Neuroplasticity in Individuals with Incomplete Spinal Cord Injury: Potential Application for Multiple Sclerosis. In: CSCI-RA 2024, Finding Commonalities and Seeking Consensus MS and SCI Rehabilitation. 2024.

[44] Sandelowski M. What’s in a name? Qualitative description revisited. Res Nurs Health. 2010;33(1):77–84. doi: 10.1002/nur.20362 20014004

[45] Whitehead AL, Sully BG, Campbell MJ. Pilot and feasibility studies: is there a difference from each other and from a randomised controlled trial?. Contemporary Clinical Trials. 2014;38(1):130–3. 24735841 10.1016/j.cct.2014.04.001

[46] Patrick CJ, Iacono WG, Venables NC. Incorporating neurophysiological measures into clinical assessments: Fundamental challenges and a strategy for addressing them. Psychol Assess. 2019;31(12):1512–29. doi: 10.1037/pas0000713 30896211 PMC6754804

[47] Lim D, Pei W, Lee JW, Musselman KE, Masani K. Feasibility of using a depth camera or pressure mat for visual feedback balance training with functional electrical stimulation. Biomed Eng Online. 2024;23(1):19. doi: 10.1186/s12938-023-01191-y 38347584 PMC10863251

[48] Eldridge SM, Chan CL, Campbell MJ, Bond CM, Hopewell S, Thabane L, et al. CONSORT 2010 statement: extension to randomised pilot and feasibility trials. BMJ. 2016;355:i5239. doi: 10.1136/bmj.i5239 27777223 PMC5076380

[49] Benn N, Heffernan M, Chan K, Lim D, Inness E, Masani K, et al. Technology Acceptance of a Novel Closed-loop Functional Electrical Stimulation Intervention from People with Stroke. Archives of Physical Medicine and Rehabilitation. 2025;106(4):e96. doi: 10.1016/j.apmr.2025.01.249

[50] Gagnon MP, Orruño E, Asua J, Abdeljelil AB, Emparanza J. Using a modified technology acceptance model to evaluate healthcare professionals’ adoption of a new telemonitoring system. Telemed J E Health. 2012;18(1):54–9. doi: 10.1089/tmj.2011.0066 22082108 PMC3270047

[51] Learmonth YC, Paul L, McFadyen AK, Mattison P, Miller L. Reliability and clinical significance of mobility and balance assessments in multiple sclerosis. Int J Rehabil Res. 2012;35(1):69–74. doi: 10.1097/MRR.0b013e328350b65f 22315143

[52] Bennett SE, Bromley LE, Fisher NM, Tomita MR, Niewczyk P. Validity and Reliability of Four Clinical Gait Measures in Patients with Multiple Sclerosis. Int J MS Care. 2017;19(5):247–52. doi: 10.7224/1537-2073.2015-006 29070965 PMC5649348

[53] Motl RW, Learmonth YC, Pilutti LA, Dlugonski D, Klaren R. Validity of minimal clinically important difference values for the multiple sclerosis walking scale-12?. Eur Neurol. 2014;71(3–4):196–202. doi: 10.1159/000356116 24457548

[54] Pilutti LA, Dlugonski D, Sandroff BM, Suh Y, Pula JH, Sosnoff JJ, et al. Further validation of multiple sclerosis walking scale-12 scores based on spatiotemporal gait parameters. Arch Phys Med Rehabil. 2013;94(3):575–8. doi: 10.1016/j.apmr.2012.08.214 22960049

[55] van Vliet R, Hoang P, Lord S, Gandevia S, Delbaere K. Falls efficacy scale-international: a cross-sectional validation in people with multiple sclerosis. Arch Phys Med Rehabil. 2013;94(5):883–9. doi: 10.1016/j.apmr.2012.10.034 23246897

[56] Nilsagård Y, Carling A, Forsberg A. Activities‐specific balance confidence in people with multiple sclerosis. Multiple sclerosis international. 2012;2012(1):613925. 22919491 10.1155/2012/613925 PMC3420074

[57] Baert I, Freeman J, Smedal T, Dalgas U, Romberg A, Kalron A, et al. Responsiveness and clinically meaningful improvement, according to disability level, of five walking measures after rehabilitation in multiple sclerosis: a European multicenter study. Neurorehabil Neural Repair. 2014;28(7):621–31. doi: 10.1177/1545968314521010 24503204

[58] Molhemi F, Monjezi S, Mehravar M, Shaterzadeh-Yazdi M-J, Majdinasab N. Validity, reliability, and responsiveness of Persian version of mini-balance evaluation system test among ambulatory people with multiple sclerosis. Physiother Theory Pract. 2024;40(3):565–75. doi: 10.1080/09593985.2022.2119908 36065714

[59] Kesar TM, Stinear JW, Wolf SL. The use of transcranial magnetic stimulation to evaluate cortical excitability of lower limb musculature: Challenges and opportunities. Restor Neurol Neurosci. 2018;36(3):333–48. doi: 10.3233/RNN-170801 29758954 PMC6106786

[60] Rossini PM, Burke D, Chen R, Cohen LG, Daskalakis Z, Di Iorio R, et al. Non-invasive electrical and magnetic stimulation of the brain, spinal cord, roots and peripheral nerves: Basic principles and procedures for routine clinical and research application. An updated report from an I.F.C.N. Committee. Clin Neurophysiol. 2015;126(6):1071–107. doi: 10.1016/j.clinph.2015.02.001 25797650 PMC6350257

[61] Hsieh H-F, Shannon SE. Three approaches to qualitative content analysis. Qual Health Res. 2005;15(9):1277–88. doi: 10.1177/1049732305276687 16204405

[62] Popovic MR, Curt A, Keller T, Dietz V. Functional electrical stimulation for grasping and walking: indications and limitations. Spinal Cord. 2001;39(8):403–12. doi: 10.1038/sj.sc.3101191 11512070

[63] Sayenko DG, et al. Neuromodulation and restoration of motor responses after severe spinal cord injury, in Cellular, Molecular, Physiological, and Behavioral Aspects of Spinal Cord Injury. Elsevier; 2022. 51–63.

[64] James ND, McMahon SB, Field-Fote EC, Bradbury EJ. Neuromodulation in the restoration of function after spinal cord injury. Lancet Neurol. 2018;17(10):905–17. doi: 10.1016/S1474-4422(18)30287-4 30264729

[65] Sayenko DG, Rath M, Ferguson AR, Burdick JW, Havton LA, Edgerton VR, et al. Self-Assisted Standing Enabled by Non-Invasive Spinal Stimulation after Spinal Cord Injury. J Neurotrauma. 2019;36(9):1435–50. doi: 10.1089/neu.2018.5956 30362876 PMC6482915

[66] Eldridge SM, Lancaster GA, Campbell MJ, Thabane L, Hopewell S, Coleman CL, et al. Defining Feasibility and Pilot Studies in Preparation for Randomised Controlled Trials: Development of a Conceptual Framework. PLoS One. 2016;11(3):e0150205. doi: 10.1371/journal.pone.0150205 26978655 PMC4792418

[67] El-Kotob R, Giangregorio LM. Pilot and feasibility studies in exercise, physical activity, or rehabilitation research. Pilot Feasibility Stud. 2018;4:137. doi: 10.1186/s40814-018-0326-0 30123527 PMC6090705

[68] Lawson DO, Mellor K, Eddy S, Lee C, Kim KH, Kim K, et al. Pilot and Feasibility Studies in Rehabilitation Research: A Review and Educational Primer for the Physiatrist Researcher. Am J Phys Med Rehabil. 2022;101(4):372–83. doi: 10.1097/PHM.0000000000001797 34091466

[69] Lancaster GA, Thabane L. Guidelines for reporting non-randomised pilot and feasibility studies. Pilot Feasibility Stud. 2019;5:114. doi: 10.1186/s40814-019-0499-1 31608150 PMC6778655

[70] Taves DR. The use of minimization in clinical trials. Contemp Clin Trials. 2010;31(2):180–4. doi: 10.1016/j.cct.2009.12.005 20060500

[71] Scott NW, McPherson GC, Ramsay CR, Campbell MK. The method of minimization for allocation to clinical trials. a review. Control Clin Trials. 2002;23(6):662–74. doi: 10.1016/s0197-2456(02)00242-8 12505244

[72] Campbell MK, Elbourne DR, Altman DG, CONSORT group. CONSORT statement: extension to cluster randomised trials. BMJ. 2004;328(7441):702–8. doi: 10.1136/bmj.328.7441.702 15031246 PMC381234

